# Case Report: Healing a Traumatic Birth

**DOI:** 10.3389/fpsyg.2020.581823

**Published:** 2020-12-22

**Authors:** Phyllis Klaus

**Affiliations:** Former Behavioral Science Instructor in the Department of Family Practice, School of Medicine, Michigan State University, East Lansing, MI, United States

**Keywords:** pregnancy, birth, trauma, breastfeeding, perinatal

## Abstract

One needs to recognize that the perinatal period is a vulnerable time where negative attitudes, words and actions can have an emotionally damaging effect on birthing women, who are especially sensitive to how they are treated, because of their high oxytocin levels. This case report depicts a traumatic birth in which the mother was needlessly separated from her baby and also harshly treated by the personnel, which had an emotionally devastating impact on the mother/baby relationship. This case report will demonstrate how psychotherapy was used to heal this distress.

## Introduction

In my work as a psychotherapist, with a specialty in perinatal problems, I am often helping people work through a traumatic or disappointing birth experience. Women are especially vulnerable during the perinatal period because the body, mind and spirit are “wide open” to be able to birth a human being physically as well as to “take in” the baby psychologically. The high levels of oxytocin contribute to this openness, which may explain why women are so susceptible to both positive support and negative experiences during this period (Uvnas-Moberg, 2019b). The following story depicts how the lack of awareness or sensitivity for a birthing mother’s emotional and physical needs and experiences caused long term negative emotional consequences for both the mother and her child.

## The Case Report

A client, pregnant with her second child, appeared for therapy because she was having disciplinary trouble with her first child, and had unresolved distress, as she had been traumatized by the unexpected events of his birth. This case demonstrates how negative experiences in connection with birth may cause a long-term negative impact on the mother’s view of the baby and her future confidence in mothering. In working with this particular client, I created a safe setting for the patient by using Active Listening and then used a combination of methods such as Age Regression, Ego State Therapy, Affect Bridge, and Reframing of Meaning (Rossi and Cheek, 1988; Cheek, 1994) in order to elicit the traumatic memories, which could then be worked with and reframed. I also used eye movement desensitization and reprocessing therapy (EMDR), a well-researched method to heal trauma (Shapiro, 2018).

## The Story

As a young girl, Mrs. M loved babies and became a favorite of neighborhood families with new babies when she offered to play with them. When she was old enough, she became a trusted babysitter. After college she became a postpartum doula and was becoming a breastfeeding consultant. Her goal was to help other women with breastfeeding in the immediate postpartum period. She always planned to breastfeed her own children one day.

She married a kind man who also believed in the importance of breastfeeding. When pregnant with their first baby, she arranged a home birth with a home birth midwife in hopes of avoiding unnecessary interventions from a hospital. She planned to have the baby skin-to-skin right after birth and looked forward to the “breast crawl” (Uvnas-Moberg, 2019c) which she had heard about from studies in Sweden, where the baby is placed on the mother’s chest and crawls and maneuvers to one of the breasts. During pregnancy, she daydreamed often about the baby breastfeeding. She talked to her prenatal baby and imagined this occurring (Uvnas-Moberg, 2019b). Unfortunately, close to the end of the pregnancy she developed high blood pressure and other significant symptoms and needed to be transferred to the hospital for an emergency cesarean section. She had no support whilst being in hospital (Trowell, 1982; Arden, 2015a) and had not planned for this possibility happening.

As soon as she recovered from the surgery, she called to the nurse and asked for her baby. She believed that even with a cesarean section she could have the baby as soon as she felt ready, and that the baby would be placed in such a way to avoid the operative area. However, unbeknownst to Mrs. M, the protocol of that hospital did not permit women to have the baby until several hours after recovery from major surgery.

She pleaded with the nurse, “Please! Please! Bring me my baby.” The nurse refused. Eventually, after Mrs. M became very agitated and upset, continuing somewhat loudly calling for her baby, the nurse said to her, “You seem to be having a psychological episode, you have to wait.” Mrs. M immediately quieted and stopped any voice pleas, fearing that if she protested maybe the nurse would call (Child Protective Services) and take the baby away. When Mrs. M eventually got the baby, she had much difficulty managing him and became angry at the baby (Trowell, 1982; Uvnas-Moberg, 2019a).

She nursed him, but it was not a rewarding experience.

Let us look at the physiology as well as the psychology of what is happening here. Oxytocin levels rise during pregnancy (Uvnas-Moberg, 2019b) and Mrs. M was further activating oxytocin release during this period with her loving thoughts and images of the baby. The emergency cesarean section caused diminished production of oxytocin (Arden, 2015a), and separation from the baby interfered with the oxytocin release normally occurring in the postpartum period (Uvnas-Moberg, 2019a). The negative treatment by the nurse also negatively impacted the release of oxytocin (Arden, 2015b).

Oxytocin levels are affected not only by labor, birth, and breastfeeding but also by kind looks, voice, and feeling being attuned to or cared about and being encouraged vs. being ignored, shamed, or frightened. These subtle events have enormous effects in particular during birth when mothers are having high oxytocin levels and become extra sensitive and open to both positive and negative environmental cues. Together the negative experiences decreased the positive effects of oxytocin normally occurring in connection with birth and consequently reducing the development of calmness, rewarding experiences and love for the newborn. In these situations, the effect pattern of oxytocin may even turn into defense and aggression against those who are experienced as intruders.

I met Mrs. M when she was pregnant with her second child and came to prepare for her second birth. She arrived with her three-year-old little boy, who was very rambunctious. She wanted to talk about her son’s birth and described what a difficult child he was. He would not mind her, he would not respond to any actions of affection, like sitting on her lap. He did not play with her, as opposed to being different at his nursery school or with his father where he acted with more ease of interaction.

## The Therapeutic Intervention

In my therapy work I created a situation in which Mrs. M felt secure and safe. One of the keys to healing is to create an atmosphere of open, thoughtful listening (Kawamichi et al., 2014). Some of the skills of Active, or Reflective Listening are useful. Open-ended statements, allow the client to fill in what they might be feeling, as opposed to direct questions. One technique is to reflect back a “feeling” related to the event which might prompt the woman to fill in more of her emotional experience. For example, “It sounds like you were feeling completely disregarded by the nurse…” Such a statement may help the woman feel understood, heard and validated.

As I took her history and listened to her story, she revealed how distressing the birth of her son had been (Uvnas-Moberg, 2019b). I wanted Mrs. M to describe what was missing for her. I asked her to close her eyes and review internally the whole event and notice all the aspects that were upsetting for her and recognize step by step all the hopes she had that were dashed (Rossi and Cheek, 1988; Cheek, 1994). Then I asked her to step into the scene and recreate in every detail what she had wanted instead and imagine that happening (Cheek, 1994). These techniques are referred to as Age Regression, Ego State Therapy, Affect Bridge and Reframing of Meaning and allow access to traumatic memories (Rossi and Cheek, 1988; Cheek, 1994). Once the trauma is accessed it can be processed, released, and healed using bi-lateral stimulation, a method developed by Francine Shapiro in EMDR therapy (Shapiro, 2018).

When she got to the memory of being angry at her little baby boy, I asked her to imagine putting another face on the baby (Batino, 2007). She immediately saw the nurse! I invited her, in her imagination, to tell the nurse what she felt and how that treatment damaged her and her feelings for her baby. I asked her to continue recreating the birth as she would have wanted it to be. After this visualization she felt much better.

She returned ten days later to plan for the second birth. Then she said something surprising: “You can’t imagine how different my little boy is. He is so loving, he wants to play with me, to sit on my lap, to hug me, to have me read him a story. It is an amazing change.”

It is interesting to notice how powerful the oxytocin effect is in response to social interaction (Uvnas-Moberg, 2019c) emanating from feelings of love and connection, as well as how sensitive oxytocin is to negative experiences, when there is perhaps a diminished oxytocin effect by seeing, hearing, and feeling negative responses. When Mrs. M asked herself, and queried to me, “why did I feel so angry at the baby?”, I reframed that experience for her by suggesting that perhaps in a profound way, directing her anger at the baby instead of projecting it at the nurse, may have been an unconscious way to save the baby from being taken away. Had Mrs. M projected her anger at the nurse, she may have been in much more trouble with the system.

There are many therapeutic methods to help heal these events. But it is especially useful with imagery to tap into the emotions, the beliefs and the body experiences of the negative event and create the potential for healing by shifting to a positive reframing of the event. If one can recreate the preferred reactions or treatment, one can start to loosen or eliminate the original negative emotional effects of the experience. Human beings are open to healing after danger has passed.

## Client’s Earlier Experiences

As a therapist, in the session or in an ensuing session, I always explore with the client any earlier thought, emotion, or experience where these emotions may have originated or been experienced. For example, if a previous event or experience may have laid the groundwork for being disregarded, the client becomes more susceptible to reacting in a similar way.

In Mrs. M’s case, she shared that the nurse’s behavior reminded her of how she was treated as a little girl, when she was trying to tell her mother that she was being abused by a cousin and her mother would not listen to her (Simkin and Klaus, 2015). She recognized that the experience of this treatment in childhood was retriggered in her interaction with the nurse and may have been an emotional underpinning to her reaction (Meyen et al., 2007; Arden, 2015b). She proceeded to tell me that she had now started talking to her mother about the past, having the voice that she had never had before. I think that it is important to notice that not only did the potential for healing and positive growth become available to Mrs. M but also for her child. She tearfully felt so positive and grateful to have made this deep understanding which helped her and her child and made a change in her life.

## Conclusion

Mrs. M’s story depicts how the cesarean section, the separation from the baby, as well as the insensitivity and a lack of awareness from the staff, affected her emotional and physical experience of birth. The nurse’s lack of training in communication skills was hurtful to Mrs. M as an adult but also triggered a childhood abuse memory (Simkin and Klaus, 2015).

In every significant event in life, humans have many layers of history and experiences that can be triggered or activated in either a positive or negative manner. Of course, the nurse did not recognize the meaning of her behavior to this mother, but regardless of her lack of knowledge, sadly the nurse’s way of handling the situation created long term pain and disturbances in the relationship between the mother and her newborn. The therapy was helpful for Mrs. M and allowed a deeper opportunity for healing.

## Ethics Statement

Written informed consent was obtained from the individual for the publication of any potentially identifiable images or data included in this article.

## Author Contributions

PK confirms being the sole contributor of this work and has approved it for publication.

## Conflict of Interest

The author declares that the research was conducted in the absence of any commercial or financial relationships that could be construed as a potential conflict of interest.
